# The double burden of severe mental illness and cancer: a population-based study on colorectal cancer care pathways from screening to end-of-life care

**DOI:** 10.1017/S2045796024000234

**Published:** 2024-05-15

**Authors:** A.-V. Seppänen, F. Daniel, S. Houzard, C. Le Bihan, M. Coldefy, C. Gandré

**Affiliations:** 1 Institut de Recherche et Documentation en Économie de la Santé (Institute for Research and Information in Health Economics – IRDES), Paris, France; 2 Institut National du Cancer (French National Cancer Institute – INCa), Boulogne Billancourt, France; 3 AP-HP, Robert Debré University Hospital, Paris, France

**Keywords:** care pathways, colorectal cancer, mental health, oncology, quality indicators, quality of care, severe mental illness

## Abstract

**Aims:**

Cancer is one of the main causes of death in persons with severe mental illness (SMI). Although their cancer incidence is similar, or sometimes even potentially lower compared to the general population, their cancer mortality remains higher. The role of healthcare provision and care equity in this mortality is increasingly being addressed in research, but available studies are limited in their scope. In this context, our aim was to compare colorectal cancer (CRC) care pathways from screening to end-of-life care in patients with and without pre-existing SMI on a national scale.

**Methods:**

This research leverages real-world data from the French national health claims database, covering the entire population, to assess cancer screening, diagnosis, treatment and post-treatment follow-up as well as quality of care (QOC) pathways among patients with incident CRC in 2015–2018, considering whether they had pre-existing SMI. We matched patients with SMI with three patients without – on age, sex, region of residence, year of cancer incidence and cancer type and location at presentation – as well as nationally established quality of CRC care indicators and regression models adjusting for relevant socio-economic, clinical and care provider-related covariates.

**Results:**

Among patients with incident CRC, 1,532 individuals with pre-existing SMI were matched with individuals without SMI. After adjusting for covariates, both colon and rectal cancer patients with SMI were less likely to participate in the national CRC screening programme and to receive advanced diagnostic examinations (e.g., colonoscopies and several complementary diagnostic examinations). They also had lower odds of receiving combined treatments (e.g., neoadjuvant chemotherapy, radiotherapy and excision) and of having access to targeted therapy or capecitabine but higher odds for invasive care (e.g., stoma). Colon cancer patients with SMI were also more likely to have no treatment at all, and rectal cancer patients with SMI were less likely to receive post-treatment follow-up. Suboptimal QOC was observed for both groups of patients, but to a higher extent for patients with SMI, with statistically significant differences for indicators focusing on diagnosis and post-treatment follow-up.

**Conclusions:**

Our findings reveal discrepancies across the care continuum of CRC between individuals with and without SMI and provide initial avenues on where to focus future efforts to address them, notably at the entry and exit stages of cancer care pathways, while calling for further research on the mechanisms preventing equity of physical healthcare for individuals with SMI.

## Introduction

Severe mental illnesses (SMI) include a cluster of mental disorders, such as schizophrenia and bipolar disorder, that cause serious functional limitations interfering with major life activities like education, work and social relationships (Cabassa, 2023). Almost 40 million persons were estimated to live with bipolar disorders and over 23 million with schizophrenia in 2019 worldwide (GBD 2019 Mental Disorders Collaborators, 2022). For these individuals, research consistently shows an excess mortality compared to the general population, with a gap that is seemingly widening (Correll *et al.*, 2022; Lawrence *et al.*, 2010; Thornicroft, 2011); life expectancy is 15–20 years shorter (Coldefy and Gandré, 2018; Nordentoft *et al.*, 2013) and, in meta-analyses, the pooled risk of all-cause mortality is over twice as high than in comparison populations regardless of the time span assessed (Correll *et al.*, 2022; Walker *et al.*, 2015), putting health equity into question (Thornicroft, 2011).

One of the main causes of death for persons with SMI is cancer (Coldefy and Gandré, 2018; Lawrence *et al.*, 2010), just like in the general population. The risk of cancer mortality is, however, higher in persons with SMI, regardless of a similar or possibly lower cancer incidence (Charlesworth *et al.*, 2023; Grassi *et al.*, 2021; Kisely *et al.*, 2013; Li *et al.*, 2018; Mahar *et al.*, 2020; Pettersson *et al.*, 2020). This suggests that factors beyond health behaviours, intervening after the onset of cancer, may play a significant role in this excess mortality. Studies have also shown that patients with SMI are more likely to have metastases (Charlesworth *et al.*, 2023; Kisely *et al.*, 2013) and more advanced stages of cancer at presentation compared to the general population (Ishikawa *et al.*, 2016), which could be partly explained by lower cancer screening rates (Gandré and Coldefy, 2020; Howard *et al.*, 2010; Solmi *et al.*, 2020; Thomsen *et al.*, 2023) and longer delays for primary care consultations (Howard *et al.*, 2010). Currently, the available literature on the role of healthcare-related factors in cancer survival also points towards less appropriate and well-organized care provision to persons with SMI at different points along the care continuum (Mahar *et al.*, 2020; Wang *et al.*, 2023). However, the evidence on care timeliness and adequacy remains scarce and inconclusive, and few studies have used comprehensive population-based data covering both community and hospital care to assess access to guideline-appropriate cancer care, beyond isolated aspects of care pathways. In the case of some common cancers, such as colorectal cancer (CRC), existing research is often restricted to limited populations (e.g., the elderly, or individuals with schizophrenia only) and rarely addresses care after diagnosis (Protani *et al.*, 2022), or solely for a few health services (e.g., emergency surgery or palliative care).

In this context, the aim of our research was to compare the quality of cancer care pathways from screening to end-of-life care in patients with and without SMI in France, using national, population-based health claims data covering community and hospital care, and a matched cohort study design. We focused on CRC, which is among the most common types of cancer and leading causes of cancer-related deaths in France (OECD, 2023), with 47,582 new cases in 2023 (Lapôtre-Ledoux *et al.*, 2023), and which has been associated with increased mortality in persons with SMI worldwide (Charlesworth *et al.*, 2023; Correll *et al.*, 2022; Cunningham *et al.*, 2015; Ishikawa *et al.*, 2016).

## Methods

### Data source

We used real-world data from the French national health claims database (*Système National des Données de Santé*, SNDS), which contains data on all care-related reimbursements made by the statutory health insurance (SHI). As France has universal healthcare coverage through the SHI, the SNDS covers the entire French population. A pseudonymized patient identifier enables linking all reimbursed outpatient care acts (consultations, medical procedures and drugs dispensed to patients), hospital stays in public and private hospitals, and information on patients’ inclusion in several state-subsidised health insurance schemes (for instance, for persons with low income or long-term illness [LTI]). The SNDS also contains basic demographic data on patients, as well as a unique identifier for each care provider. Persons with long-term conditions, such as SMI and cancer, are identified via the standardized Healthcare Expenditures and Conditions Mapping (HECM) (*Cartographie des Pathologies et des Dépenses*) tool, which combines causes for hospitalizations, reasons for entering the LTI scheme, dispensed drugs and medical procedures to identify these conditions (Rachas *et al.*, 2022).

### Study population

Our study population included all men and women, 18 years of age or older; treated for incident colon, rectal or both cancers (double location) (International Classification of Diseases [ICD-10] codes C18 [except C181], C19, C20); without or with lymph-node involvement (C775); and without or with synchronous metastases (C77 [except C775], C78, C79). To obtain a sufficient sample size for analysing the impact of SMI in different subgroups of CRC patients, we included SHI beneficiaries with incident CRC over a period of 4 years (2015–2018). We did not include incident cases from 2019 onwards, as cancer screening and care were strongly affected by the COVID-19 pandemic (CNAM, 2022a). Having CRC a given year was defined through the HECM tool as being hospitalized or included in the LTI scheme for CRC, or having CRC-specific drugs or medical procedures. Incidence was defined by an absence of hospitalizations and LTI inclusions for CRC in the 5 preceding years, and an absence of CRC-specific drugs and medical procedures in the preceding 365 days. To avoid heterogeneity in care pathways, we excluded patients treated for other cancers concomitantly or in the year before CRC incidence, as well as patients with in situ CRC (ICD-10 codes D01.0, D01.1 and D01.2) for the analysis of cancer care pathways.

We then identified CRC patients with pre-existing SMI, i.e., having SMI in the year preceding cancer incidence. Included conditions were, in accordance with international literature (Liu *et al.*, 2022), schizophrenia, schizotypal and delusional disorders (ICD-10 codes F20-F29) as well as manic episodes and bipolar affective disorders (ICD-10 codes F30 and F31). Having SMI a given year was defined through the HECM tool as being included in the LTI scheme for SMI that year, or hospitalized for SMI in the past 2 years, or hospitalized for SMI in the past 5 years while still receiving recurrent antipsychotic drug treatment, i.e., at least three deliveries of such treatments over the year – including most drugs with Anatomical Therapeutic Chemical codes starting by N05 and lithium (CNAM, 2022b). To take into account the cyclic nature of SMI, patients *not* identified with an SMI but with a recurrent antipsychotic drug treatment in the year prior to cancer incidence, were considered to have pre-existing SMI if they had SMI in the year of cancer incidence.

### Study design

To obtain comparable groups of CRC patients with and without SMI in terms of demographics and cancer characteristics, we adopted a matched cohort study design using exact matching with replacement. We matched each patient with SMI to three patients without SMI based on age (±5 years), sex, region of residence, year of cancer incidence, cancer location (colon, rectum or both) and cancer type at presentation (invasive non-metastatic without lymph-node involvement, invasive non-metastatic with lymph-node involvement and synchronous metastatic). Patients with onset of SMI after CRC diagnosis were excluded from the pool of potential matches.

### Cancer care pathways and quality indicators

Data on care pathways included, depending on the cancer type and location, participation in the national CRC screening programme (faecal immunochemical test) within 12 months before cancer incidence, main diagnostic examinations received and their total number, treatments and treatment combinations received, as well as post-treatment follow-up.

To assess the quality of CRC care pathways, we used nationally established quality of care (QOC) indicators developed by the French National Cancer Institute (*Institut National du Cancer*, INCa), under the umbrella of the French national health authority (*Haute autorité de santé*, HAS). The indicators cover diagnosis and cancer staging, timeliness of treatments, 90-day postoperative mortality, post-treatment follow-up and end-of-life care, and assess the proportion of patients having received care according to established recommendations for their specific type of cancer. Two thresholds define the level of QOC for each indicator: a *target threshold*, such as the ideal proportion of patients that should be receiving a colonoscopy before their first treatment (≥90%), and an *alert threshold* raising concern for care quality, for instance if less than 80% of patients receive a colonoscopy before their first treatment (INCa, 2022). The original thresholds for postoperative mortality and post-treatment follow-up were not applicable to our study, as they were initially constructed for comparing mortality and follow-up between hospitals and not populations. The 90-day postoperative mortality indicator, originally reflecting hospital mortality, was adapted to reflect mortality in our study population.

### Covariates

We included one comprehensive clinical covariate: the mortality-related morbidity index (MRMI), which is a quantified measure of overall morbidity, specifically developed for the SNDS data, and based on the presence of the most common chronic diseases. These include cardiovascular and respiratory disorders, diabetes, chronic renal failure, multiple sclerosis, paraplegia, epilepsy, dementia, Parkinson’s disease, liver and pancreatic disorders, inflammatory bowel diseases and substance use disorders (Constantinou *et al.*, 2018). This index was adapted to our study by excluding SMI and cancers from the potential comorbidities (modified MRMI). While the use of this index is optimal within a population that has not been selected on a given condition, it still demonstrates a higher performance than other widely used indexes (Elixhauser and Charlson) for individuals with specific disorders in the SNDS data (CNAM, 2021).

Socio-economic covariates included patients’ inclusion in the publicly subsidised complementary health insurance (*Couverture Maladie Universelle Complémentaire*, CMU-C), or in the voucher plan for the purchase of a complementary health insurance (*Aide à l’acquisition d’une Complémentaire Santé*, ACS), which were combined into a single variable used as a proxy for low income, as they are only available for persons with limited earnings.

Care provider-related covariates included the type of the main hospital providing CRC care (public general hospital, public teaching hospital, non-profit comprehensive cancer centre – *Centre de Lutte Contre le Cancer*, CLCC –, private non-profit hospital or private-for-profit hospital). To take into account geographical differences in care provision, we used a measure of local potential accessibility to general practitioners (GPs) calculated at the patient’s residential zip code (Barlet *et al.*, 2012) as well as the density of gastroenterologists and hepatologists in the county (*département*) of residence at the year of cancer incidence (DREES, 2021), which were both used as continuous variables to avoid information loss. At an individual level, we also assessed whether each patient had a referring physician (*médecin traitant*) reported in the year of cancer incidence.

### Statistical analyses

First, we compared the sociodemographic and clinical characteristics of all CRC patients with and without SMI prior to matching, using Chi^2^ and Wilcoxon tests. After matching, we assessed differences between patients with and without SMI in participation in the national CRC screening programme (among eligible patients; aged 50–74 years) and receipt of main diagnostic examinations, treatments and post-treatment follow-up, for colon and rectal cancer and invasive non-metastatic and metastatic cancer separately where relevant. We then assessed whether QOC thresholds were attained and estimated differences in QOC indicators between the two groups. For this, we used simple and multivariable conditional logistic regression models for binary variables or conditional Poisson regressions for count variables, taking into account correlation between matched patients, and adjusting for relevant socio-economic, clinical and care provider-related covariates: low income and MRMI (all models), having a referring physician (for screening), local potential accessibility to GPs (for screening and diagnosis), density of gastroenterologists and hepatologists in the county of residence (for diagnostic and follow-up colonoscopy) and type of hospital providing CRC care (for treatment and follow-up).

## Results

**Figure 1.**
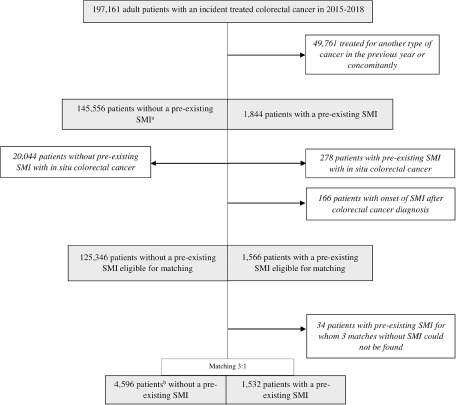
Study flow chart.

^a^SMI: Severe mental illness; ^b^4,499 individuals were selected through matching with replacement, of whom 91 (2.0%) matched twice and 3 (0.07%) matched three times.

A total of 147,400 patients had incident CRC without other concomitant cancers in 2015–2018 (Fig. 1). Among these patients, 1,844 (1.3%) had pre-existing SMI. Patients with SMI were more likely to be younger, female and to have low income, a higher number of comorbidities and colon cancer compared to patients without SMI (Table 1). For each type of cancer, patients with SMI had higher rates of metastatic cancer at presentation. After excluding patients with in situ CRC, 1,532 patients with pre-existing SMI were matched with 3 patients without SMI (Fig. 1). Matches could not be found for 34 (2.2%) patients with SMI (Fig. 1 and Supplementary Table S1).

**Table 1.** Characteristics of colorectal cancer patients with and without pre-existing SMI (before matching)

	Patients with SMI (*n* = 1,844)	Patients without SMI (*n* = 145,556)
	Mean (±SD) or n (%) or *Median* [IQR]	
**Sociodemographic characteristics**		
Age (years)*	*67.0* [59.0–76.0]	*70.0* [62.0–79.0]
Sex (female)*	968 (52.5)	63,995 (44.0)
Inclusion in CMU-C or ACS schemes for persons with low income*	352 (19.1)	8,775 (6.0)
*N* missing values^a^	3 (0.2)	55 (0.04)
**Year of cancer incidence**		
2015	457 (24.8)	35,474 (24.4)
2016	489 (26.5)	40,203 (27.6)
2017	432 (23.4)	34,923 (24.0)
2018	466 (25.3)	34,956 (24.0)
**Clinical characteristics**		
MRMI comorbidity index*	1.24 (±1.44)	0.81 (±1.13)
**Cancer type by location**		
Colon*		
In situ	233 (17.7)	15,842 (16.4)
Invasive non-metastatic^b^ without lymph-node involvement	791 (60.0)	58,081 (60.0)
Invasive non-metastatic^b^ with lymph-node involvement	76 (5.6)	7,582 (7.8)
Synchronous metastatic	219 (16.6)	15,313 (15.8)
**Rectum**		
In situ	33 (7.6)	3,447 (8.5)
Invasive non-metastatic^b^ without lymph-node involvement	289 (66.3)	27,807 (68.3)
Invasive non-metastatic^b^ with lymph-node involvement	33 (7.6)	3,662 (9.0)
Synchronous metastatic	81 (18.6)	5,784 (14.2)
**Colon and rectum**		
In situ	12 (13.5)	755 (9.4)
Invasive non-metastatic^b^ without lymph-node involvement	35 (39.3)	4,085 (50.8)
Invasive non-metastatic^b^ with lymph-node involvement	4 (4.5)	433 (5.4)
Synchronous metastatic	38 (42.7)	2,765 (34.4)

Abbreviations: SD: standard deviation; IQR: interquartile range; CMU-C: couverture maladie universelle complémentaire (publicly subsidised complementary health insurance); ACS: Aide à l’acquisition d’une Complémentaire Santé (voucher plan for the purchase of a complementary health insurance); MRMI: mortality-related morbidity index.

a Values missing at the year of cancer incidence were completed with data retrieved from the preceding year, when available.

b Or metachronous metastatic.

* *p* < 0.05 for the difference between the two groups with and without SMI.

Differences in cancer care between matched patients with and without SMI were observed for patients with both non-metastatic and metastatic CRC. After matching and adjusting for clinical, socio-economic and care provider-related covariates, colon cancer patients with SMI were less likely to participate in the national CRC screening programme (adjusted odds ratio [aOR] = 0.80; 95% confidence interval [95%CI]: 0.65–0.99) and to receive colonoscopy (aOR = 0.69; 95%CI: 0.59–0.81), and had a lower average number of recommended diagnostic examinations (aOR = 0.95; 95%CI: 0.92–0.99) compared to patients without SMI (Table 2). Regarding treatment, they were less likely to receive excision by endoscopy (aOR = 0.84; 95%CI: 0.73–0.97) as well as any chemotherapy (aOR = 0.71; 95%CI: 0.63–0.82) and targeted therapy (aOR = 0.80; 95%CI: 0.65–1.00), but more likely to have stoma (aOR = 1.42; 95%CI: 1.12–1.79). Regarding treatment combinations, they were less likely to receive both excision and chemotherapy (aOR = 0.80; 95%CI: 0.69–0.93) and more likely to receive open surgery only (aOR = 1.24; 95%CI: 1.08–1.43) or no treatment at all (aOR = 2.76; 95%CI: 1.51–5.04).

**Table 2.** Cancer care received by matched colon cancer patients with and without SMI, and odds for patients with SMI to receive each care item compared to patients without SMI

	**Invasive non-metastatic**^a^		Metastatic		**Invasive non-metastatic**^a^ **and metastatic**			
	SMI (*N* = 861)	Non-SMI (*N* = 2,583)	SMI (*N* = 213)	Non-SMI (*N* = 639)	Univariable analysis		**Multivariable analysis**^b^	
	n (%) or *median* [IQR]		n (%) or *median* [IQR]		OR (SMI vs non-SMI)	95%CI	aOR (SMI vs non-SMI)	95%CI
**Cancer screening**								
Participation in organized screening within 12 months before first cancer treatment (50–74 years old only)	117 (22.7)	500 (31.4)	11 (8.0)	65 (15.6)	0.62*	[0.51–0.76]	0.80*	[0.65–0.99]
**Diagnostic tests received**								
Colonoscopy	601 (69.8)	1,969 (76.2)	133 (62.4)	471 (73.7)	0.69*	[0.60–0.80]	0.69*	[0.59–0.81]
CT scan for abdomen and pelvis	698 (81.1)	2,079 (80.5)	199 (93.4)	582 (91.1)	1.07	[0.89–1.29]	0.99	[0.81–1.21]
CT scan for chest	488 (56.7)	1,578 (61.1)	143 (67.1)	419 (65.6)	0.87	[0.76–1.01]	0.88	[0.76–1.02]
CT scan for chest, and abdomen and pelvis	481 (55.9)	1,540 (59.6)	141 (66.2)	414 (64.8)	0.89	[0.78–1.03]	0.90	[0.78–1.04]
Number of diagnostic tests received (out of colonoscopy, CT scan for abdomen and pelvis, and CT scan for chest)	*3* [1–3]	*3* [1–3]	*2* [2–3]	*3* [2–3]	0.96*	[0.92–0.99]	0.95*	[0.92–0.99]
**Treatment procedures received**								
Excision by endoscopy	353 (41.0)	1,281 (19.6)	33 (15.5)	139 (21.8)	0.71*	[0.62–0.81]	0.84*	[0.73–0.97]
Excision by open surgery	731 (84.9)	2,172 (84.1)	166 (77.9)	433 (67.8)	1.20	[1.00–1.44]	1.08	[0.89–1.32]
Any chemotherapy^c^	171 (19.9)	647 (25.1)	138 (64.8)	529 (82.8)	0.70*	[0.62–0.80]	0.71*	[0.63–0.82]
*Oral chemotherapy (Capecitabine)*^d^	40 (4.7)	142 (5.5)	20 (9.4)	68 (10.6)	0.85	[0.63–1.15]	0.91	[0.67–1.25]
Targeted therapy^d^	14 (1.6)	51 (2.0)	76 (35.7)	292 (45.7)	0.77*	[0.63–0.94]	0.80*	[0.65–1.00]
Stoma or derivation	91 (10.6)	155 (6.0)	47 (22.1)	105 (16.4)	1.68*	[1.35–2.09]	1.42*	[1.12–1.79]
**Treatment combinations received**								
Excision by endoscopy only	105 (12.2)	370 (14.3)	0 (0.0)	3 (0.5)	0.83	[0.66–1.03]	1.07	[0.85–1.35]
Excision by open surgery (with or without endoscopy) only	565 (65.6)	1,546 (59.9)	66 (31.0)	91 (14.2)	1.38*	[1.21–1.57]	1.24*	[1.08–1.43]
Chemotherapy only	3 (0.4)	16 (0.6)	29 (13.6)	144 (22.5)	0.59*	[0.41–0.84]	0.57*	[0.38–0.84]
Excision and chemotherapy	163 (18.9)	620 (24.0)	105 (49.3)	352 (55.1)	0.77*	[0.67–0.89]	0.80*	[0.69–0.93]
Other	6 (0.7)	14 (0.5)	6 (2.8)	39 (6.1)	0.68	[0.36–1.27]	0.63	[0.33–1.21]
None	19 (2.2)	17 (0.7)	7 (3.3)	10 (1.6)	2.94*	[1.70–5.07]	2.76*	[1.51–5.04]
**Follow-up**								
Number of CT scans within 3 years after last surgery^e^	*4* [3−6]	*5* [3−6]	NA	NA	0.92	[0.80–1.06]	0.96	[0.84–1.11]
Between 1 and 2 complete colonoscopies within 3 years after last surgery^e^	35 (57.4)	197 (70.6)	NA	NA	0.56	[0.31–1.00]	0.64	[0.35–1.15]

Abbreviations: IQR: interquartile range; (a)OR: (adjusted) odds ratio; 95%CI: 95% confidence interval; CT: computed tomography; NA: not applicable.

Note: all analyses exclude patients with colon and rectal cancer (double location).

a Or metachronous metastatic.

b Adjusting for clinical, socio-economic and care provider-related covariates

c Intravenous or oral (capecitabine).

d Adjuvant or neoadjuvant.

e Among patients with invasive non-metastatic colon cancer who had cancer surgery in combination with chemotherapy and/or radiotherapy, and with data available at least 39 months after last surgery (maximum until 2021). Excluding patients with Crohn’s disease, ulcerative colitis, Lynch’s syndrome or familial adenomatous polyposis, or who developed metastatic cancer, had recurrence of cancer, moved abroad or deceased during the follow-up period. Sample size: *N* = 61 and *N* = 279 for patients with and without SMI, respectively).

* *p* < 0.05

Findings were similar for patients with SMI and rectal cancer (Table 3). They were less likely to participate in the national CRC screening programme (aOR = 0.59; 95%CI: 0.42–0.84) and to receive colonoscopy (aOR = 0.68; 95%CI: 0.50–0.93). Patients with SMI also had a lower average number of diagnostic examinations (aOR = 0.92; 95%CI: 0.87–0.97) and were less likely to receive CT scans (aORs between 0.72 and 0.77) and pelvic MRI (aOR = 0.78; 95%CI: 0.62–1.00). Regarding treatment, they had lower odds of receiving radiotherapy (aOR = 0.69; 95%CI: 0.53–0.90) as well as chemotherapy (e.g., any chemotherapy: aOR = 0.61; 95%CI: 0.49–0.76, and capecitabine: aOR = 0.57; 95%CI: 0.42–0.76). Regarding treatment combinations, patients with SMI were less likely to receive a combination of neoadjuvant chemotherapy, radiotherapy and excision (aOR = 0.68; 95%CI: 0.47–0.98), and more likely to receive open surgery only (aOR = 1.38; 95%CI: 1.08–1.75). Regarding post-treatment care, they were less likely to receive follow-up CT scans (aOR = 0.73, 95%CI: 0.59–0.90).

**Table 3.** Cancer care received by matched rectal cancer patients with and without SMI, and odds for patients with SMI to receive each care item compared to patients without SMI

	**Invasive non-metastatic**^a^		Metastatic		**Invasive non-metastatic**^a^ **and metastatic**			
	SMI (*N* = 316)	Non-SMI (*N* = 948)	SMI (*N* = 76)	Non-SMI (*N* = 228)	Univariable analysis		Multivariable analysis^b^	
	n (%) or *median* [IQR]		n (%) or *median* [IQR]		OR (SMI vs non-SMI)	95%CI	aOR (SMI vs non-SMI)	95%CI
**Cancer screening**								
Participation in organized screening within 12 months before first cancer treatment (50–74 years old only)	41 (18.6)	204 (30.7)	7 (12.5)	27 (16.2)	0.55*	[0.39–0.77]	0.59*	[0.42–0.84]
**Diagnostic tests received**								
Colonoscopy	258 (81.7)	808 (85.2)	55 (72.4)	193 (84.7)	0.69*	[0.52–0.93]	0.68*	[0.50–0.93]
CT scan for abdomen and pelvis	239 (75.6)	747 (78.8)	64 (84.2)	209 (91.7)	0.78	[0.59–1.03]	0.72*	[0.54–0.97]
CT scan for chest	195 (61.7)	613 (64.7)	51 (67.1)	170 (74.6)	0.85	[0.67–1.07]	0.77*	[0.59–0.99]
CT scan for chest, and abdomen and pelvis	191 (60.4)	609 (64.2)	51 (67.1)	167 (73.3)	0.83	[0.66–1.05]	0.76*	[0.60–0.98]
Pelvic MRI	131 (41.5)	431 (45.5)	30 (39.5)	133 (58.3)	0.76*	[0.60–0.95]	0.78*	[0.62–1.00]
Number of diagnostic tests received (out of colonoscopy, CT scan for abdomen and pelvis, CT scan for chest, and pelvic MRI)	*3* [1–4]	*3* [2–4]	*3* [2–4]	*3* [3–4]	0.93*	[0.88–0.98]	0.92*	[0.87–0.97]
**Treatment procedures received**								
Excision by endoscopy	161 (51.0)	500 (52.7)	19 (25.0)	64 (28.1)	0.92	[0.74–1.15]	1.03	[0.81–1.29]
Excision by open surgery	242 (76.6)	774 (81.7)	45 (59.2)	152 (66.7)	0.74*	[0.57–0.95]	0.78	[0.60–1.03]
Radiotherapy	92 (29.1)	323 (34.1)	18 (23.7)	94 (41.2)	0.71*	[0.55–0.91]	0.69*	[0.53–0.90]
Any chemotherapy^c^	98 (31.0)	413 (43.6)	60 (79.0)	201 (88.2)	0.62*	[0.50–0.76]	0.61*	[0.49–0.76]
*Neoadjuvant chemotherapy*^c^	58 (18.4)	268 (28.3)	14 (18.4)	85 (37.3)	0.52*	[0.40–0.69]	0.55*	[0.41–0.73]
*Adjuvant chemotherapy*^c^	44 (13.9)	221 (23.3)	31 (40.8)	118 (51.8)	0.58*	[0.46–0.75]	0.63*	[0.48–0.82]
*Oral chemotherapy (Capecitabine)*^d^	60 (19.0)	280 (29.5)	12 (15.8)	57 (25.0)	0.56*	[0.42–0.75]	0.57*	[0.42–0.76]
Targeted therapy^d^	4 (1.3)	22 (2.3)	30 (39.5)	105 (46.1)	0.78	[0.56–1.09]	0.77	[0.55–1.08]
Stoma or derivation	77 (24.4)	212 (22.4)	17 (22.4)	60 (26.3)	1.05	[0.80–1.37]	0.95	[0.72–1.25]
**Treatment combinations received**								
Excision by endoscopy only	46 (14.6)	130 (13.7)	0 (0.0)	1 (0.4)	1.06	[0.75–1.51]	1.20	[0.82–1.75]
Excision by open surgery (with or without endoscopy) only	144 (45.6)	361 (38.1)	8 (10.5)	8 (3.5)	1.39*	[1.1–1.74]	1.38*	[1.08–1.75]
Chemotherapy only	3 (1.0)	4 (0.4)	19 (25.0)	32 (14.0)	1.88*	[1.17–3.04]	1.30	[0.74–2.26]
Neoadjuvant chemotherapy, radiotherapy and excision	41 (13.0)	165 (17.4)	3 (4.0)	17 (7.5)	0.69*	[0.49–0.98]	0.68*	[0.47–0.98]
Excision and adjuvant chemotherapy	22 (7.0)	112 (11.8)	19 (25.0)	51 (22.4)	0.73	[0.52–1.02]	0.78	[0.54–1.12]
Excision, adjuvant chemotherapy and radiotherapy	7 (2.2)	11 (1.2)	2 (2.6)	6 (2.6)	1.60	[0.73–3.54]	1.38	[0.61–3.10]
Other	47 (14.9)	157 (16.6)	22 (29.0)	106 (46.5)	0.74*	[0.55–0.99]	0.75	[0.55–1.02]
None	6 (1.9)	8 (0.8)	3 (4.0)	7 (3.1)	1.82	[0.79–4.16]	1.55	[0.66–3.67]
**Follow-up**								
Number of CT scans within 3 years of last surgery^e^	*4* [2–5]	*5* [3–6]	NA	NA	0.74*	[0.60–0.92]	0.73*	[0.59–0.90]
Between 1 and 2 complete colonoscopies within 3 years after last surgery^e^	22 (56.4)	120 (75.5)	NA	NA	0.42*	[0.20–0.85]	0.50	[0.22–1.11]

Abbreviations: IQR: interquartile range; SD: standard deviation; (a)OR: (adjusted) odds ratio; 95%CI: 95% confidence interval; CT: computed tomography; MRI: magnetic resonance imaging; NA: not applicable.

Note: all analyses exclude patients with colon and rectal cancer (double location).

a Or metachronous metastatic.

b Adjusting for clinical, socio-economic and care provider-related covariates.

c Intravenous or oral (capecitabine).

d Adjuvant or neoadjuvant.

e Among patients with invasive non-metastatic colon cancer who had cancer surgery in combination with chemotherapy and/or radiotherapy, and with data available at least 39 months after last surgery (maximum until 2021). Excluding patients with Crohn’s disease, ulcerative colitis, Lynch’s syndrome or familial adenomatous polyposis, or who developed metastatic cancer, had recurrence of cancer, moved abroad or deceased during the follow-up period. Sample size: *N* = 39 and *N* = 159 patients with and without SMI, respectively).

* *p* < 0.05.

Regarding the QOC indicators, none of the target thresholds were attained regardless of SMI status, but patients with SMI were systematically farther from attaining them (Table 4). For patients with SMI, all but two indicators on timeliness between diagnosis and treatment and end-of-life-care (Table 4), were within the alert range, raising concern for the quality of their care pathways. For patients without SMI, all but three indicators – also on care timeliness and end-of-life care (Table 4) – were within the alert range. After adjusting for covariates, statistically significant differences between patients with and without SMI were found for indicators focusing on diagnosis and cancer staging as well as post-treatment follow-up. Patients with SMI and non-metastatic colon cancer were significantly less likely to have a complete diagnostic workup and cancer staging before their first treatment (aOR = 0.80; 95%CI: 0.66–0.96) and patients with SMI and non-metastatic colon, rectal or both cancers were less likely to have postoperative CT scans and colonoscopies within 3 years after their last surgery (aOR = 0.40; 95%CI: 0.22–0.75) compared to patients without SMI (Table 4).

**Table 4.** Indicators of the quality of cancer care pathways in patients with and without pre-existing SMI

			SMI	Non-SMI	Univariable analysis		**Multivariable analysis**^a^	
	Target threshold	Alert threshold	% (*n*/*d*)	% (*n*/*d*)	OR (SMI vs non-SMI)	95%CI	aOR (SMI vs non-SMI)	95%CI
**Diagnosis and cancer staging**								
Patients with **non-metastatic colon** cancer who had a complete or partial colonoscopy within 6 weeks before their first treatment^b^	≥90%	<80%	52.2% (352/674)	64.6% (1,351/2,092)	0.51*	[0.51–0.71]	0.65*	[0.54–0.79]
Patients with **non-metastatic colon** cancer who had a chest, and abdominal and pelvic CT scan within 6 weeks before their first treatment^b^	≥90%	<80%	49.7% (335/674)	55.7% (1,166/2,092)	0.66*	[0.66–0.93]	0.92	[0.77–1.11]
*Patients with **non-metastatic colon** cancer who had a complete diagnostic workup and cancer staging*^c^ *before their first treatment*^b^	≥90%	<80%	35.5% (239/674)	44.4% (929/2,092)	0.69*	[0.58–0.82]	0.80*	[0.66–0.96]
Patients with **non-metastatic rectal** cancer who had a complete or partial colonoscopy before their first treatment^b^	≥90%	<80%	76.9% (170/221)	82.6% (571/691)	0.48	[0.48–1.01]	0.72	[0.49–1.05]
Patients with **non-metastatic rectal** cancer who had a chest, and abdominal and pelvic CT scan before their first treatment^b^	≥90%	<80%	61.1% (135/221)	66.1% (457/691)	0.60	[0.60–1.08]	0.81	[0.59–1.10]
Patients with **non-metastatic rectal** cancer who had a pelvic MRI before their first treatment^b^	≥90%	<80%	20.4% (45/221)	26.6% (184/691)	0.49	[0.49–1.02]	0.78	[0.53–1.14]
Patients with **non-metastatic rectal** cancer who had a rectal echo-endoscopy before their first treatment^b^	≥90%	<80%	16.3% (36/221)	18.5% (128/691)	0.57	[0.57–1.28]	0.89	[0.58–1.39]
*Patients with **non-metastatic rectal** cancer who had a complete diagnostic workup and cancer staging*^d^ *before their first treatment*^*b*^	>90%	<75%	14.9% (33/221)	20.0% (138/691)	0.72	[0.48–1.08]	0.74	[0.49–1.14]
**Treatment timeliness**								
Patients with **non-metastatic colon** cancer who had their first treatment^b^ within 6 weeks after their last biopsy^e^	>90%	<75%	64.0% (240/375)	67.2% (896/1,334)	0.87	[0.68–1.11]	1.03	[0.80–1.34]
Patients with **non-metastatic rectal** cancer who had their first treatment^b^ within 8 weeks after their last biopsy^e^	>90%	<75%	73.4% (105/143)	75.9% (352/464)	0.89	[0.58–1.36]	0.87	[0.55–1.37]
Patients with **metastatic colon, rectal or both** cancers who had their first treatment^b^ within 8 weeks after their last biopsy^e^	>90%	<75%	83.2% (99/119)	88.3% (371/420)	0.66	[0.38–1.17]	0.69	[0.38–1.27]
Patients with **non-metastatic rectal** cancer who had neoadjuvant chemotherapy and radiation therapy simultaneously^f^^,^^g^	>70%	<50%	36.4% (8/22)	43.3% (45/104)	0.59	[0.21–1.66]	0.60	[0.18–1.99]
**Postoperative mortality (adapted indicators)**								
90-day postoperative mortality in patients with **non-metastatic colon** cancer^h^	NA^i^	NA^i^	6.0% (33/553)	3.1% (52/1,672)	1.98*	[1.26–3.11]	1.27	[0.74–2.18]
90-day postoperative mortality in patients with **non-metastatic rectal** cancer^h^	NA^i^	NA^i^	1.7% (4/232)	1.2% (7/604)	1.50	[0.43–5.24]	0.97	[0.19–5.03]
90-day postoperative mortality in patients with **non-metastatic colon or rectal** cancer who *had urgent surgery*^h^	NA^i^	NA^i^	19.5% (22/113)	12.2% (16/131)	1.59	[0.82–3.08]	1.35	[0.67–2.73]
**Post-treatment follow-up**								
Patients with **non-metastatic colon, rectal or both** cancers who had a minimum of 6 chest, and abdominal and pelvic CT scans within 3 years after their last surgery^j^	NA^i^	NA^i^	26.9% (28/104)	40.1% (178/444)	0.55*	[0.34–0.88]	0.56*	[0.33–0.94]
Patients with **non-metastatic colon, rectal or both** cancers who had between 1 and 2 complete colonoscopies within 3 years after their last surgery^j^	NA^i^	NA^i^	55.8% (58/104)	72.5% (322/444)	0.47*	[0.30–0.73]	0.59*	[0.37–0.93]
*Patients with **non-metastatic colon, rectal or both** cancers who had a minimum of 6 chest, and abdominal and pelvic CT scans and 1–2 complete colonoscopies within 3 years after their last surgery*^j^	NA^i^	NA^i^	15.4% (16/104)	32.0% (142/444)	0.38*	[0.22–0.68]	0.40*	[0.22–0.75]
**End-of-life care**								
Patients with metachronous or synchronous **metastatic colon, rectal or both** cancers who had chemotherapy during their last month of life^k^	<15%	>30%	17.4% (47/270)	24.3% (167/686)	0.65*	[0.45–0.95]	0.70	[0.47–1.04]

Abbreviations: *n*: numerator; d: denominator; (a)OR: (adjusted) odds ratio; 95%CI: 95% confidence interval; CT: computed tomography; MRI: magnetic resonance imaging.

Note: Metachronous metastatic cancers were considered non-metastatic unless otherwise stated.

a Adjusting for clinical, socio-economic and care provider-related covariates.

b Calculated among patients who had chemotherapy, radiotherapy or open surgery. Excluding patients who had their first radiotherapy in the private sector, whose first treatment was urgent surgery, and patients for whom a first treatment was not identified. or oral chemotherapy. For indicators “Patients with non-metastatic colon cancer who had a complete diagnostic workup and cancer staging before their first treatment” and “Patients with non-metastatic rectal cancer who had a complete diagnostic workup and cancer staging before their first treatment”: patients whose first treatment was oral chemotherapy were also excluded.

c Complete or partial colonoscopy, biopsy, and chest, and abdominal and pelvic CT scan within 6 weeks before first treatment.

d Complete or partial colonoscopy, biopsy, and chest, abdominal and pelvic CT scan within 8 weeks before first treatment, and pelvic MRI within 4 weeks before first treatment.

e Calculated among patients who had a biopsy.

f Intravenous chemotherapy within ±5 days or oral chemotherapy (capecitabine) within ±7 days of neoadjuvant radiation therapy.

g Calculated among patients who had cancer surgery and radiotherapy, excluding patients who had radiotherapy in the private sector.

h Mortality regardless of cause. Calculated among patients who had cancer surgery and included in the General Health Insurance scheme (*regime general*) only. Excluding patients with Crohn’s disease or ulcerative colitis.

i As the indicators were originally developed to assess QOC at the hospital level, the thresholds for postoperative mortality and follow-up were not applicable to our study.

j Among patients with invasive non-metastatic colon cancer who had cancer surgery in combination with chemotherapy and/or radiotherapy, and with data available at least 39 months after last surgery (maximum until 2021). Excluding patients with Crohn’s disease, ulcerative colitis, Lynch’s syndrome or familial adenomatous polyposis, or who developed metastatic cancer, had recurrence of cancer, moved abroad or deceased during the follow-up period.

k Calculated among deceased patients with metachronous or synchronous metastatic colon, rectal or both cancers.

* *p* < 0.05

## Discussion

Based on exhaustive population-based data and a matched cohort study design, our findings reveal discrepancies in CRC care pathways from screening to post-treatment follow-up between patients with and without SMI. We consistently found less participation in the national CRC screening programme, less advanced diagnostic examinations as well as a lower likelihood of receiving certain treatment combinations and capecitabine or targeted therapy in patients with SMI, for both colon and rectal cancer. Differences were also found in post-treatment follow-up, but only for patients with rectal cancer. None of the QOC target thresholds were attained, with patients with SMI being farther from attaining them, and most indicators were within the alert range for both patients with and without SMI. This raises questions about care organization, quality and practices, even though underreporting of certain procedures may occur, requiring caution when interpreting the results. All in all, significant differences were found for QOC indicators focusing on diagnosis and post-treatment follow-up, but not for other indicators.

Our findings underscore that discrepancies in CRC care for patients with SMI already start at the entry stage of care pathways and in particular screening, while it is free of charge in France. Previous research suggests that many of the reasons for non-uptake of cancer screening are similar in persons with SMI and the general population, although reported more frequently in persons with SMI, such as practical barriers (e.g., no transport, lack of formal reminders) or embarrassment (Howard *et al.*, 2010). In addition, concerns directly related to SMI may be competing with preventive care (including screening) and less acute physical health concerns, especially if the SMI is exacerbated, or coexisting with other chronic conditions (Bhatia *et al.*, 2021; Jensen *et al.*, 2015).

Discrepancies were further observed in diagnostic examinations received: patients with SMI were less likely to have colonoscopy, several other recommended diagnostic examinations, and a complete diagnostic workup and cancer staging before their first treatment. This is consistent with research on other types of cancer, such as breast cancer, where women with SMI were found to have a lower likelihood of receiving the recommended diagnostic examinations compared to women without SMI (Seppänen *et al.*, 2023). Diagnostic overshadowing by healthcare providers, i.e., attributing somatic complaints to SMI, is hypothesized to contribute to disparities in diagnosis (Howard *et al.*, 2010; Thornicroft, 2011). This could lead to diagnostic examinations being delayed until the situation becomes more acute or to cancer only being discovered during examinations for other conditions. It is thus possible that some of the colon cancer cases were discovered during emergency surgery, for instance for occlusion, which is why colonoscopies where not performed. Yet, colonoscopy rates remain the lowest in the patients with SMI even after positive faecal occult blood tests (Bhatia *et al*., 2022). Qualitative research and other reports from care providers point out difficulties to perform invasive procedures on patients with SMI, including greater challenges in preparing them for colonoscopy (Thomsen *et al.*, 2023), and stigmatizing perceptions related to their mental disorder, such as undesired behaviour at the hospital, or tendencies to miss medical appointments, which can influence care-related decisions (Gandré *et al.*, 2023; Grassi and Riba, 2021; Howard *et al.*, 2010; Thornicroft, 2011; Tuesley *et al.*, 2019).

Along the cancer care continuum, our results also demonstrate disparities in CRC treatment for patients with SMI. They were less likely to receive certain care combinations (excision and chemotherapy for colon cancer, and neoadjuvant chemotherapy, radiotherapy, and excision for rectal cancer), and to have access to targeted therapy or capecitabine compared to patients without SMI, but more likely to receive invasive forms of treatment such as stoma. Our results are congruent with international research on colorectal and other cancers (Ishikawa *et al.*, 2016; Kisely *et al.*, 2013; Protani *et al.*, 2022) and complement our previous findings on breast cancer (Seppänen *et al.*, 2023), reproducing results on a large sample of both men and women. The observed differences in treatments may stem from a range of factors. More invasive treatments may result from more advanced cancer at presentation, which we were only able to assess using the subtype of cancer at diagnosis. However, other studies have shown that even when taking cancer stage into account, differences in care approaches remain (Ishikawa *et al.*, 2016; Wang *et al.*, 2023). An increased risk of complications related to cancer treatments, for instance due to drug interactions with antipsychotic medications, could also conduct care providers to avoid some treatment options, such as chemotherapy, for patients with SMI (Glasdam *et al.*, 2023; Howard *et al.*, 2010). Furthermore, certain cancer treatments, such as radiotherapy, may cause distress in patients with paranoia, hallucinations or severe anxiety, due to the clinical set-up (restrained movement and being unaccompanied during the treatment), in which case surgical excision might be preferable (Howard *et al.*, 2010). Adverse effects from certain treatments can also be considered too severe for a person with SMI to manage alone, especially in the case of social isolation (Gandré *et al.*, 2023).

Moreover, significant discrepancies between patients with and without SMI were found for some of the post-treatment follow-up variables, consistent with our previous findings on breast cancer (Seppänen *et al.*, 2023). These findings could reflect the siloed nature of physical and mental healthcare, that has been highlighted in a number of national contexts (Gandré *et al.*, 2023; Irwin *et al.*, 2017), and which could be an important limiting factor for effective transitional and follow-up care. However, statistically significant differences between patients with and without SMI regarding post-treatment follow-up were not found for all variables and population subgroups included in our analyses.

Finally, our results underscore the need to improve the overall quality of cancer care pathways for both patients with and without SMI. None of the QOC target thresholds were attained, and most indicators were within the alert range, indicating that the provision of recommended CRC diagnosis, treatment and post-treatment procedures may be suboptimal. Although patients with SMI were systematically farther from attaining the thresholds, we only found statistically significant differences between the two groups for QOC indicators focusing on the entry (diagnosis) and exit (post-treatment follow-up) stages of care pathways. Regardless of resorting to data on the whole French population, our analyses may be underpowered to detect differences in the smaller subpopulations included in the calculation of some of the QOC indicators and should be interpreted with caution. Thus, similar disparities may exist along the rest of the care pathways, but that our study was underpowered to detect. In addition, differences compared to the general population should not be the only indicator to consider; in their absence, major systemic QOC improvements may still be necessary (McGinty *et al.*, 2012), requiring particular efforts to avoid exacerbating inequities, as health- and care-related improvements in vulnerable populations often lag behind those in the general population (Solmi *et al.*, 2020).

Our findings should be interpreted in light of several limitations. The SNDS is based on health claims data and therefore only allows identifying persons with SMI that resort to the healthcare system. Furthermore, persons that do not seek care may also have undiagnosed or late diagnosis of cancer. Our findings therefore provide a conservative estimate of differences in cancer care between persons with and without SMI. The SNDS data also contain limited individual clinical and sociodemographic data. Thus, we did not have access to cancer stage at presentation, health-related behaviours (smoking, body mass index, etc.) and detailed socio-economic factors (such as profession, country of origin and family situation) that may impact access, timeliness and use of healthcare services and health outcomes (Cabassa, 2023; Gandré *et al.*, 2023; Loretti, 2021; OECD, 2023). It is additionally possible that some healthcare procedures, such as CT scans, are underreported, leading to an underestimation of their use. However, our aim was to compare care between patients with and without SMI, for whom potential underreporting is unlikely to be different. The SNDS also does not contain data on participation in therapeutic patient education programmes nor in supportive cancer care, not always covered by the SHI but increasingly part of care recommendations (NHS England, 2016). Moreover, although matching is a robust technique for group comparisons, some patients with SMI could not be matched to three patients without SMI, and were consequently excluded from our analyses. However, these patients only represented 2% of individuals with SMI and incident cancer, which is likely to have limited impact on our results. Finally, we were not able to assess the reasons for non-receipt of some health services along the CRC care pathways. Therefore, we cannot conclude directly on whether discrepancies in care between patients with and without SMI stem from supply-side (provider-related) or demand-side (patient-related) factors. Nevertheless, a complementary qualitative approach to this research points to a role of both (Gandré *et al.*, 2023), while we were able to identify where inequities emerge along the cancer care pathways, which can inform interventions designed to reduce inequities in healthcare that should notably target the entry and exit stages of care pathways where most disparities were found. Our analyses may have been underpowered to detect all potential discrepancies in care pathways, and we were unable to perform adjusted analyses in subpopulations, such as by type of cancer and sex. Further research using larger sample sizes is needed for assessing potential discrepancies these subpopulations, notably in countries with more inhabitants, since we already used exhaustive data for France.

All in all, despite limitations, our study was based on comprehensive population-based health claims data covering the whole French population and allowing for the identification of an appropriate control group, as well as on nationally established QOC indicators covering all phases of CRC care pathways. This allows providing novel knowledge on discrepancies in guideline-concordant care in patients with SMI in real-world practice while yielding good external validity. Our findings can therefore be used to inform clinical practice, policy-making and further research.

## Conclusions

Our findings reveal discrepancies across the CRC care continuum in France for individuals with and without SMI: patients with SMI are in particular more likely to have less treatment combinations and less targeted therapy but more invasive care than patients without SMI, while diagnoses and post-treatment follow-up processes are not guideline-concordant. These results highlight the need for further studies on the mechanisms preventing equity of physical healthcare in this population and provide avenues on where to focus efforts in future interventions and practice aiming to improve the quality of cancer care for patients with SMI.

## Supporting information

Seppänen et al. supplementary material 1 Seppänen et al. supplementary material

Seppänen et al. supplementary material 2 Seppänen et al. supplementary material

## Data Availability

This study used data from the SNDS database, which can under no circumstances be shared according to French law. Aggregated data can be made available by the corresponding author upon request.

## Supplementary material

The supplementary material for this article can be found at https://doi.org/10.1017/S2045796024000234.
