# Supplementary material for: The double burden of severe mental illness and cancer: a population-based study on colorectal cancer care pathways from screening to end-of-life care
Source: Epidemiol Psychiatr Sci. 2024 May 15;33:e27. doi: 10.1017/S2045796024000234 (PMC11362684; doi:10.1017/S2045796024000234)
Supplement: Seppänen et al. supplementary material 1 — Seppänen et al. supplementary material [file S2045796024000234sup001.pdf]

Supplementary Table 1. Characteristics of matched and unmatched colorectal cancer patients with pre-existing SMI

	**Matched patients** (n=1,532)	**Unmatched patients** (n=34)
	Mean (±SD) or n (%) or *Median* [IQR]	
**Sociodemographic characteristics**		
**Age (years)***	*68.0* [60-77]	*61.5* [43-80]
**Sex (female)**	818 (53.4)	19 (55.9)
**Inclusion in CMU-C or ACS schemes for persons with low income**	301 (19.7)	8 (23.5)
**Year of cancer incidence**		
2015	392 (25.6)	11 (32.4)
2016	406 (26.5)	8 (23.5)
2017	359 (23.4)	7 (20.6)
2018	375 (24.5)	8 (23.5)
**Clinical characteristics**		
**MRMI comorbidity index**	1.31 (±1.47)	0.97 (±1.27)
**Cancer type by location**		
**Colon***		
Invasive non-metastatic† with lymph-node involvement	71 (6.6)	5 (41.7)
Invasive non-metastatic† without lymph-node involvement	790 (73.6)	1 (8.3)
Synchronous metastatic	213 (19.8)	6 (50.0)
**Rectum***		
Invasive non-metastatic† with lymph-node involvement	28 (7.1)	5 (45.5)
Invasive non-metastatic† without lymph-node involvement	288 (73.5)	1 (9.1)
Synchronous metastatic	76 (19.4)	5 (45.5)
**Colon and rectum***		
Invasive non-metastatic† with lymph-node involvement	1 (1.5)	3 (27.3)
Invasive non-metastatic† without lymph-node involvement	33 (50.0)	2 (18.2)
Synchronous metastatic	32 (48.5)	6 (54.6)

Abbreviations: SD: standard deviation; IQR: interquartile range; CMU-C: couverture maladie universelle complémentaire (publicly subsidised complementary health insurance); ACS: aide à l'acquisition d'une complémentaire santé (voucher plan for the purchase of a complementary health insurance); MRMI: mortality-related morbidity index

Note: *$p < 0.05$ for the difference between the matched and non-matched groups

† Or metachronous metastatic
